# A case with high bilirubinemia and hemolytic anemia during leptospirosis and a short review of similar cases

**DOI:** 10.22088/cjim.11.4.441

**Published:** 2020

**Authors:** Edmond Puca, Erjona Abazaj, Pellumb Pipero, Arjan Harxhi, Redi Ferizaj, Najada Como, Entela Puca

**Affiliations:** 1 Service of Infection Diseases, University Hospital Center, Tirana, Albania; 2 Institute of Public Health, Tirana, Albania; 3 Valley Baptist Medical Center, 2101 Pease Street Harlingen, TX 78550, USA; 4 Service of Endocrinology, American Hospital, Tirana, Albania

**Keywords:** Leptospirosis, Weil’s syndrome, Hyperbilirubinemia, Hemolytic anemia

## Abstract

**Background:**

Leptospirosis is characterized by very diverse clinical manifestations, which may range from flu-like subclinical forms to very severe presentations characterized by multi-organ failure, or to atypical presentations. One of its most aggressive presentations is Weil’s disease, characterized by jaundice, hemorrhagic phenomena and renal failure. Cases with high bilirubinemia over 30mg/dL are not communes in human leptospirosis. Our aims are to present an atypical case presentation of human leptospirosis, characterized by jaundice and hemolytic anemia, and to make a short review in PubMed for similar cases. At the same time we want to emphasize the diversity of the clinical presentation of human leptospirosis.

**Methods:**

A 54-year-old man presents at the emergency department of the infectious medicine with severe fatigue, nausea, vomiting, and generalized weakness. On exam, he was alert and well oriented; blood pressure was 80/50 mmHg and icteric. First blood examinations confirmed high bilirubinemia, thrombocytopenia and acute renal failure.

**Results:**

Based on anamnestic and clinical evaluations, blood and serology examinations, the patient resulted with leptospirosis. The bilirubin reached 73.4mg/dL. At the same time on PubMed research we found only limited cases with leptospirosis associated with bilirubinemia over 30mg/dL and over less with hemolytic anemia.

**Conclusion:**

Based on our clinical experience, as well as literature data, we suggest that clinicians should have a high index of suspicion in cases of jaundice with exposure possibilities for infectious diseases. Connection of high bilirubinemi over then 30mg/dL and hemolytic anemia in human leptospirosis is an unical case report.

Leptospirosis is a tropical infectious disease caused by pathogenic spirochetes of genus Leptospira. Mammals are the most widespread reservoirs for it and the pathogens are transmitted directly or indirectly from animals to humans. Usually people are infected through contact with materials or products contaminated by the excretions of rodents (1, 2). Risk factors are related to professional activity, but other factors such as gender are not excluded (1–3). This illness is characterized by a very diverse clinic manifestations, which may range from flu-like subclinical forms to severe presentations characterized by multi-organ failure, or to atypical presentations with some key symptoms presenting, and others missing (4,5). One of its most aggressive presentations of Leptospirosis is Weil’s syndrome, characterized by jaundice, hemorrhagic phenomena and renal failure. In the last decade, in our service, we have had 20-30 cases of leptospirosis annually (2). Cases with high bilirubinemia over 30mg/dL are not communes in human leptospirosis (6–9). Even less is the presence of hemolytic anemia (10). In this presentation we want to present a case of atypical presentation of the disease, with Weil’s syndrome characterized by hyperbilirubinemia and hemolytic anemia.

Our patient a male, reported muscle pain, fatigue, decreasing urine. He was icteric and has blood pressure was 80/50 mmHg at the presentation of hospital. Based on clinical presentation, epidemiologic data and then serological examination, he resulted with leptospirosis. 

Most leptospirosis cases are mild and resolve spontaneously. In all suspected cases of leptospirosis, is necessary to start treatment of antimicrobial and supportive therapy. The patient was treated with ampicillin, fresh frozen plasma, blood transfusions and other support therapy. Anemia resolved soon after discharge, but bilirubin continues to be elevated and only returned to normal about 45 days after admission. The patient is followed for two months after discharged. Through this publication we want to emphasize the diversity of the clinical presentation of human leptospirosis. We have also reviewed in PubMed for Weil’s syndrome literature cases presenting with hyperbilirubinemia or hemolytic anemia for the last ten years. 

## Case Presentation

A 54-year-old man presents at the emergency department with severe fatigue, nausea, vomiting, and generalized weakness. He reported muscle pain, fatigue, and weakness at work for 4-5 days and had had a decrease in the amount of urine produced in the last two days. On examination, blood pressure was 80/50 mmHg. The patient was alert and well oriented. There was a post-operative scar on the right flank. His sclera was icteric, mucous membranes somewhat dry, skin with preserved turgor and elasticity. Two days before admission, he was seen by rheumatologist for severe lower back pain, and thought to have sciatica. For this pain he underwent a CT of the lumbar spine, which showed no abnormalities to explain his symptoms. The patient lived in the outskirts of the city and was currently involved in construction work (concrete breakage in collapsed buildings). He had undergone two surgical interventions (surgery two years ago for right pyelo-urethral pathology and another for inguinal hernia one year ago), but had no chronic diseases. Two issues though – hypotension and oliguria – although not glaring, raised our suspicion for leptospirosis.

Management: The patient is hospitalized and fluids started to hydrate the patient. He is catheterized and despite aggressive hydration for the next 12 hours, the patient continued with anuria. Therapy consisted of ampicillin 6gr/day, fluids, and fresh frozen plasma (PNF). After reaching blood pressure of 110/70, patient started on furosemide, but again still remained anuric for another 36 hours. In the following few days, diuresis finally began. Despite the fact that the renal, diuretic and platelet ratios had a tendency to improve, bilirubin continued to grow, and on the fourth day of hospitalization reached 73.4mg/dL. In the following days the clinical situation of the patient continued to be guarded. Irrespective of diuresis, the patient was tired and very icteric. There were hemorrhagic elements in the gingival, nose, and a nevus on his arm, which we needed to pack to stop the bleeding. During the days D3-D6, the patient continued to be anxious and agitated and had elements of encephalopathy. 

**Table 1:** Laboratory examinations for the patient follow-up

	D0	D1	D2	D3	D4	D5	D6	D7	D10	D13	D21	D25	D33
WBCc 4-10×103/mm	12.1	6.6	8.8	16.4	12.2	15.8	14.2	13.4	13.2	5.6	4.3	5.4	.9.4
Eritrocite 4-2-601×10/mm	3.83	3.40	2.98	3.45	3.62	3.43	2.99	2.51	2.72	2.21	2.45	3.33	3.54
Hemoglobine 11-16.5g/dl	11.2	10.4	10.1	10.1	10.3	9.9	8.8	7.5	8.2	6.5	7.4	9.2	11.0
Hematocrite 35.9-50.0L%	36.9	33.2	26.5	31	34.7	30.8	28.1	22.8	23.4	21.4	23.8	32.0	34.4
Platclets 150-400x10/mm	42	71	72	43	82	74	134	204	379	726	378	286	409
UREA 10-43 mg/dl	179	213	281	362	357	363	351	284	100	42	47	45	41
Creatincmia 0.6-1.4mg/dl	4.8	5.1	7.0	7.3	5.2	4.6	4.6	2.8	1.4	1.1	1.2	0.9	1.0
AST 0-35U/L	240	182	145	115	84	77	81	100	88	44	37	71	37
ALT 0-45UL	164	161	158	160	139	113	104	107	153	103	69	67	60
LDH 125-250UL	432	384	363	602	758	625	581	581	450	273	240	257	220
CPK	1450	1334	401	149	111	128	136	135	52	34	45	51	
Tot.bibinubin 0.3-1.2mg/dl	32.9	31.8	40.8	50.9	73.4	68.7	54.7	42.7	40.8	11.9	7.6	7.3	3.6
Dircect bilirubin 0.3-1.4mg/dl	21.0		22.1		32.5		27.3				3.5		1.8
Total protein 6.2-8.3g/dl	6/0	5.1	5.3	5.7	5.3	5.5	5.1	4.6	4.1	5.3	6.4	6.7	7.4
Albmin 3.5-5.2g.dl	3/0	2.4	2.4	2.7	2.8	2.7	2.6	2.5	2.9	3.4	3.7	4.1	3.9

Serologic results showed ELISA IgM positive and IgG negative for leptospira. The patient resulted negative for: hepatitis B and C; hemorrhagic fevers (CCHF, HFRS); chronic diseases. We did not perform hemorrhagic fever with renal syndrome or PCR. The polyuria started on the third day, lasted for 5 days and the largest amount of diuresis resulted 12L/24 hours. He had progressively worsening anemia and on the seventh day was hemotransfused. Findings of hemolysis included a reticulocyte increase. The Coomb tests were negative. The clinical situation was improving, except for the progressively worsening anemia. Regardless of the absence of major hemorrhagic phenomena or melena, patient still needed another unit of hemotransfusion on the thirteenth day. 

**Fig 1:**
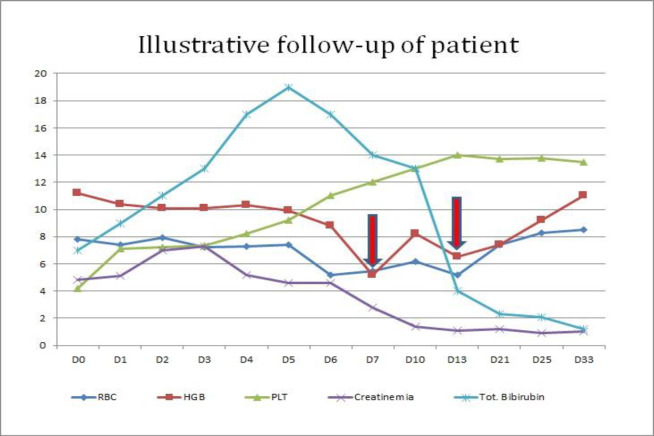
Illustrative follow-up of patient

Anemia and the overall clinical picture improved in the following few days, and the patient was discharged in stable condition, but still icteric. The patient was followed for two months. Anemia resolved soon after discharge, but bilirubin continues to be elevated and only returned to normal about 45 days after admission. 

**Literature review: **We queried PubMed with key words: human hyperbilirubinemia, Weil’s syndrome. The search resulted in 229 papers. We then selected the cases in which they were published in English. Non-English papers included: German 7, undetermined language 4, French 3, and Turkish, Serbian, Russian, Spanish and Croatian 1 each. After the first selection, only 43 publications were to be evaluated. Among the reviewed publication, there were 4 published papers in which bilirubin was ≥30mg/dL.

**Discussion and Conclusions:** Leptospirosis is an endemic disease in our country, especially after the rainy season (2). Risk factors for infection are related to exposure to leptospira, which highlights the importance of a thorough occupational and recreational history. In this case, revisiting the history was critical to reaching the diagnosis. It is possible our patient could have been infected by direct contact contamination of his food, from contact with their mice or urine, either in his apartment or as a consequence of his occupation in demolishing old buildings. Leptospirosis is characterized by clinical variation (1-10). Following incubation, which lasts about a week, nonspecific symptoms and findings such as fever, loss of appetite, nausea and vomiting are observed in 90% of cases, and are mostly confused with other bacterial or viral infections. It is often a disease that is not diagnosed by a clinician. The case we are presenting contains some unusual presentations of the disease. 

First of all, the way the disease started. Based on clinical data, and in our previous publications, fever is a significant part of leptospirosis presentation. In this case however, the patient never reported the fever. Two days before being hospitalized, the patient was consulted by the rheumatologist for low back pain. Usually low back pain is not one of the main complaints of leptospirosis. Myalgia on the other hand is one of the more common complaints. Patient only came to the infectious disease doctor due to the development of jaundice.

Second, another important element was the highly elevated bilirubin value. The presence of jaundice is characteristic in Weil’s disease, but such high values are not often presented in literature. From the research we did in PubMed, there were only four publications where the bilirubin resulted to be higher than 30mg/dL. Pothuri et al. and Covic et al reported, apart from each other, from one case with bilirubin over 30 mg/dl (6, 7). Sing et al. reported a case of HEV infection and leptospirosis (8). Legris et al. report a case of leptospirosis and amebiasis in which the bilirubin was over 29.23mg/dL (9). Jaundice is an important manifestation of hepatic dysfunction, but its mechanism in leptospirosis remains not completely elucidated. Hepatocellular damage and disruption of hepatocyte intercellular junctions leads to leakage of bile from bile canaliculi to sinusoidal blood vessels, which accounts for elevated levels of direct bilirubin seen in icteric forms of leptospirosis. But as seen in our patient's laboratory evaluations, the values of the hepatic injury indicators, liver enzymes were not particularly elevated, so we think that hyperbilirubinemia in this case was mostly associated with the blockage of bile ducts. 

Third, another atypical part of the presentation was the severe anemia. In the early days of the disease it is common to develop thrombocytopenia, together with a slight decrease in hematocrit and hemoglobin levels. Our patient was characterized by epistaxis, gastrointestinal bleeding, and hemorrhage from a mole in his arm, but this was not massive hemorrhage to explain the anemia that developed. Even platelets were in satisfactory levels. Hemolytic anemia is a rare complication of Weil's disease, but its mechanisms are still not clear. Solmazgul et al. report of a severe acute hemolytic anemia complicating leptospirosis (10). Some others suggest phospholipase activity hemolysins as the cause of leptospirosis linked hemolytic anemia. Leptospira hemolysins have been implicated in the pathogenesis of leptospirosis; however, its role as a virulence factor and/or the mechanism of action has not been fully understood (11). The mortality due to leptospirosis varies from 1-20%. It is dependent on many factors including: serovar, the clinical spectrum, complications, comorbidities, nutritional status and old ages. Fatal outcome is mainly related to renal failure although other features such as hyperkalaemia, thrombocytopenia, cardiovascular failure with hypotension, arrhythmia, respiratory failure associated with hemoptysis, neurological manifestations, gastrointestinal bleeding, repeated nausea and vomiting, contribute to the mortality rate (12). Based on these indicators our patients presented: renal failure, thrombocytopenia, hypotension, encephalopathy, hyperbilirubinemia and gastrointestinal disturbance.

On the limitations of this case, is that we did not perform specific test for leptospirosis like MAT or PCR, but only ELISA in two separately time. However, based on clinical data, epidemiological history, laboratory and serological examinations, including differential diagnosis, we concluded that we were before a case with leptospirosis.

As conclusions, the clinical manifestations of leptospirosis are quite varied. Identification of occupational activities that put patients at risk of exposure to contaminated water or infected animals such as farmers, construction workers, is very important. Based on our clinical experience, as well as literature data, we suggest that clinicians should have a high index of suspicion in cases of jaundice with exposure possibilities for infectious diseases. 

**Abbreviations**

CT- computerized axial tomography

PNF- fresh frozen plasma

ELISA- enzyme-linked immunosorbent assay

CCHF- Crimean-Congo haemorrhagic fever

HFRS- hemorrhagic fever with renal syndrome

D- day MAT- microscopic agglutination test

PCR- real-time PCR 

'Declarations 

The manuscript contains all the following sections
